# A System of Medicine

**Published:** 1900-04

**Authors:** 


A SYSTEM OF MEDICINE. Edited by F. Clifford Allbutt, M.D., F.R.C.P., etc., etc. Pp. 998. Price $5.00. Published by Macmillan.

The eighth and last volume of this excellent system is now being distributed to subscribers, who, we are sure, must be well satisfied with their purchase. This volume concludes nervous diseases, and considers mental diseases and diseases of the skin. Among those who write on nervous diseases are Drs. Vivian Poore, Risien Russell, MacKenzie, Ormerod, Tuke, Manson, Horsley, Allbutt, Chiene, and Sir Wm. Gowers. In the section on Mental Diseases, most of the best known British authorities have a part, and the subjects considered include such matters as the dullness and nervousness of children, vice and crime, all the well-marked forms of insanity and mental weakness, as well as laws relating to lunacy. Diseases of the skin occupy not quite half the volume, and the names of many of the authors are well known on this side of the Atlantic. They include Drs. Colcott Fox, Payne, Brooke, Abraham, MacKenzie, Pringle, Radcliffe Crocker, Jonathan Hutchinson, Shield, Malcolm Morris, and others. The editor is to be congratulated on the happy accomplishment of what must have been an exceedingly onerous task. A. W. S.

---

SURGICAL PATHOLOGY AND THERAPEUTICS. By John Collins Warren, M.D., LL.D., Professor of Surgery in Harvard University. Second Edition. Published by W. B. Saunders, Philadelphia. Price, Cloth, $5.00 net; Half Morocco, $6.00.

For many years the great work of Billroth on Surgical Pathology held undisputed domain in the physician's library, and justly so. It is still a great work, but since even its last edition and the death of its distinguished author, much advancement has been made in this domain of pathology. Dr. Warren has given to the profession a thoroughly scientific and masterful treatise on this ever interesting subject. In this new edition an appendix has been added "containing an enumeration of the scientific aids to surgical diagnosis, together with a series of sections on regional bacteriology." This last addition is exceedingly valuable, and we must say adds materially to the worth of the book. The chromolithographs are beautiful, and the microscopic appearances of the various bacteria are beautifully produced. The arrangement of the different subjects is well chosen. It was but natural to expect a great work from the pen of such a distinguished surgeon as Dr. Warren, and this volume will remain standard for many years to come.

A POCKET MEDICAL DICTIONARY, giving the Pronunciation and Definition of the Principal Words used in Medicine and the Collateral Sciences, including very complete Tables of Clinical Eponymic Terms of the Arteries, Muscles, Nerves, Bacteria, Bacilli, Micrococci, Spirilla, and Thermometric Scales, and a Dose-List of Drugs and their Preparations, in both the English and Metric Systems of Weights and Measures. By George M. Gould, A.M., M.D. Fourth Edition, revised and enlarged, 30,-000 words. Published, 1900, by P. Blakiston's Son & Co., 1012 Walnut St., Philadelphia. Price $1.00.

The profession is already familiar with Gould's Dictionaries and appreciates their worth. Their sale has been enormous, attesting their value. The fourth edition has been carefully revised, enlarged and now fully meets every requirement of a medical word-book.

---

THE AMERICAN YEAR-BOOK OF MEDICINE AND SURGERY. A A Yearly Digest of Scientific Progress and Authoritative Opinion in all branches of Medicine and Surgery, drawn from Journals, Monographs and Text-Books, of the leading American and Foreign Authors and Investigators, collected and arranged with critical Editorial Comment by fifteen Collaborators, under the Editorship of Dr. George M. Gould. Published 1900, in two volumes, by W. B. Saunders, Philadelphia. Price, cloth, $3.00; in half morocco, $3.75 per volume.

The Year-Book is now published in two volumes, devoted to medicine and surgery respectively. The convenience of this arrangement, in view of the unhandy bulk of previous issues, is apparent. The work contains a wealth of information, and to one who wishes to keep abreast of the progress of the times, it is indispensable. The volumes are unstintingly illustrated, well printed, well bound and exhaustively indexed.

---

A MANUAL OF THE PRACTICE OF MEDICINE. Prepared especially for Students. By A. A. Stevens, A.M., M.D. Fifth Edition. Revised and Enlarged. Philadelphia. W. B. Saunders. Price, $2.00 net.

It is well for every practitioner to possess one or more manuals to read with an eye to keeping up with the current of medical thought and progress. Stevens is a good one for this purpose. It is brief but comprehensive, and is very readably written. It is not intended to take the place of more ambitious works, but it has a clearly defined sphere of usefulness.

---

A MANUAL OF MODERN SURGERY. An Exposition of the Accepted Doctrines and Approved Operative Procedures of the Present Time. For the Use of Students and Practitioners. By John B. Roberts, A.M., M.D., Professor of Anatomy and Surgery in the Philadelphia Polyclinic, etc. Second Edition, Revised and Enlarged. Illustrated with 473 Engravings and Eight Plates in Colors and Monochrome. Lea Brothers & Co., Philadelphia and New York.

This book, already popular through the success of its first edition, is a very practical treatise on surgery. It is clearly and concisely written, all extraneous matter being carefully eliminated. It is especially adapted to the student who wishes to get at the pith of the subject without having to wade through a mass of verbiage. The plates are good and sufficiently numerous and the typography excellent. The second edition, including advances in surgery since the first issue, brings the book quite up to date.

---

MATERIA MEDICA AND THERAPEUTICS. An Introduction to the Rational Treatment of Disease, for the Use of Students and Practitioners of Medicine. By J. Mitchell Bruce, M.D., F.R.C.P., etc., Physician and Lecturer on Medicine at Charing Cross Hospital, London. New (6th) Edition, Revised and Enlarged. In one 12mo volume of 618 pages. Cloth, $1.50, net. Lea Brothers & Co., Philadelphia and New York. 1899.

Bruce's Materia Medica and Therapeutics still remains one of the most popular handbooks on the subject, and has for years been employed as a text-book in many of the leading medical colleges. The last edition has been brought up to date and the most important of the newer remedies receives sufficient consideration.

---

**This Journal and Atlanta Semi-weekly Journal one year for $1.00 cash in advance. No checks.**

THE PRINCIPLES OF TREATMENT AND THEIR APPLICATIONS TO PRACTICAL MEDICINE. By J. Mitchell Bruce, M.A., M.D., F.R.C.P., Professor and Lecturer on the Principles and Practice of Medicine, Charing Cross Hospital, etc. Adapted to the United States Pharmacopœia by E. Quin Thornton, M.D., Demonstrator of Therapeutics, Pharmacy, and Materia Medica in the Jefferson Medical College, Philadelphia. Lea Brothers & Co., Philadelphia and New York, 1900.

This is one of the most admirable books of the year, and destined to be one of the most popular. The treatment of the subject matter is especially commendable. The clinical side of doctor's work is described and gone through with from beginning to end. The various modes of treatment are detailed with a clearness that brings the patient very close to the gaze of the reader. The author's vast experience and knowledge of materia medica speaks from every page. As a valuable guide and ready and efficient help the book cannot be too highly recommended.

---

***NOTICE.—This JOURNAL and THE INTERNATIONAL JOURNAL OF SURGERY one year for $1.00 cash, to new subscribers. No out-of-town checks accepted unless 10c. is added for cost of cashing.***

---

### SHEER SELFISHNESS.

"Her husband died of heart failure."

"Oh, dear, how selfish! Why couldn't he have been ill a couple of months or so, that she might have had a chance at that lovely young doctor nearby. But some men are so thoughtless."—*Ex.*

---

**This Journal and Atlanta Semi-weekly Journal one year for $1.00 cash in advance. No checks.**

---

### HE WAS AN EXPANSIONIST.

"As the statement of an abstract scientific truth," soliloquized the physician, "it is strictly correct to say that cold contracts. But there are exceptions." It was the height of the influenza season, and he was looking at his bank account.—*Chicago Tribune.*

